# Application of Diffusion Kurtosis Imaging and Histogram Analysis for Assessing Preoperative Stages of Rectal Cancer

**DOI:** 10.1155/2018/9786932

**Published:** 2018-06-05

**Authors:** Hui Xie, Guangyao Wu

**Affiliations:** Department of Radiology, Zhongnan Hospital of Wuhan University, Wuhan, 430071 Hubei, China

## Abstract

**Objective:**

To explore the value of diffusion kurtosis imaging (DKI) and histogram analysis for assessing preoperative stages and heterogeneity in rectal cancer.

**Methods:**

Fifty patients with pathologically confirmed rectal adenocarcinoma were enrolled. The value of DKI parameters and histogram metrics for assessing the preoperative stages and heterogeneity in rectal cancer was analyzed retrospectively.

**Results:**

(1) ADC-10th percentile and ADC-25th percentile were significantly higher in T1-2 than in the T3-4 rectal cancer (the ADC values were 0.65 ± 0.08 × 10^−3^ mm^2^/s versus 0.58 ± 0.11 × 10^−3^ mm^2^/s and 0.73 ± 0.11 × 10^−3^ mm^2^/s versus 0.65 ± 0.11 × 10^−3^ mm^2^/s; *p* values were 0.035 and 0.024, resp.). (2) *D*-10th percentile and *D*-25th percentile were also significantly higher in T1-2 than in T3-4 rectal cancer (the *D* values were 0.96 ± 0.19 × 10^−3^ mm^2^/s versus 0.84 ± 0.16 × 10^−3^ mm^2^/s and 1.15 ± 0.27 × 10^−3^ mm^2^/s versus 0.99 ± 0.18 × 10^−3^ mm^2^/s; *p* values were 0.017 and 0.044, resp.). (3) *K* value and its histogram metrics showed no statistically significant difference between T1-2 and T3-4. (4) *D*-10th had the largest area under the curve (AUC 0.799) among all the parameters; the sensitivity and specificity were 84.2 and 61.3%, respectively. (5) DKI combined with traditional MRI had an accuracy of 68% while assessing the lymph node of rectal cancer.

**Conclusion:**

DKI parameters and histogram metrics are rather valuable in assessing the preoperative stages of rectal cancer; *D*-10th percentile exhibits the highest diagnostic efficiency.

## 1. Introduction

Rectal cancer is one of the most common malignant tumors in the gastrointestinal tract, which accounts for approximately 50%–70% of colorectal cancers. With the advances in the society, economic status, and changes in people's diet, the incidence of rectal carcinoma has increased gradually in the world [1, 2]. Thus, making early and accurate staging to rectal cancer, a vital problem when selecting an appropriate therapeutic method and lowering recurrence, is essential. Magnetic resonance imaging (MRI) examination has the characteristics of noninvasive, multiparameter, and multisequence, which make it the first choice when assessing rectal cancer. Diffusion kurtosis imaging (DKI) was first reported by Jensen et al. [3] in 2005, which provided the microstructure and pathophysiological information of tissues. However, only a few DKI studies with respect to rectal cancer in preoperative staging are yet available, such as prostate cancers, breast cancers, and gliomas [4–6]. Thus, the present study explored the application of DKI and its histogram for assessing the preoperative stages and heterogeneity of rectal cancer.

## 2. Materials and Methods

### 2.1. Patients

From March 2016 to April 2017, the patients with pathologically confirmed adenocarcinoma were selected at our hospital. The inclusion criteria for the patients were as follows. MRI examinations were fulfilled including multiple *b* values, DKI sequences, and conventional T1WI/T2WI sequences. The radical surgical resection and pathology staging results were clear (reference criteria AJCC Cancer Staging Manual 7th edition) [7]. The exclusion criteria were as follows: (a) MRI examination was conducted after hormonal or radiation treatment; (b) interval between the MRI and surgery was >2 weeks [8]; (c) only local surgery without radical resection was performed; and (d) images had severe artifacts. Thus, a total of 50 patients were included in the study, consisting of 27 men (54%) and 23 women (46%), at a mean age of 57.7 ± 11.7 (range, 29–86) years.

### 2.2. MRI Protocols

The MRI examinations were performed using a 3.0 T MR scanner (Magnetom Prisma, Siemens Healthcare, Erlangen, Germany) with a dedicated 32-channel pelvic phased-array coil to optimize signal-to-noise ratio. Before MRI, all the patients underwent bowel cleaning. A dose of 20 mg of the spasmolytic agent hyoscine butylbromide (Buscopan, Boehringer Ingelheim) was administered intravenously to all patients immediately to minimize bowel peristalsis and avoid motion artifacts. The MR sequences consisted of the oblique axis (vertical intestine) T1-weighted imaging, oblique axis T2-weighted imaging, and oblique axis DKI sequences (with *b* values of 200, 500, 1000, 1500, and 2000 s/mm^2^). The detailed scan parameters were as follows: oblique axis T2WI: TR (time of repetition) = 3770 ms, TE (time of echo) = 101 ms, PAT (parallel acquisition technique) = 2, FOV (field of view) = 200 × 200 mm^2^, matrix size = 128 × 128, slice thickness = 4 mm, and number of sections = 20; oblique axis T1WI: TR = 700 ms, TE = 12 ms, PAT = 2, FOV = 280 × 280 mm, matrix size = 128 × 128, slice thickness = 4 mm, and number of sections = 20; and DKI: TR = 4900 ms, TE = 87 ms, flip angle = 90°, PAT = 4, FOV = 280 × 280 mm^2^, matrix size = 128 × 128, voxel size = 1.1 × 1.1 × 5 mm, slice thickness = 4 mm, number of sections = 20, and acquisition time = 260 s. In addition, the largest *b* value of DKI was 2000 s/mm^2^ [9]. The MRI protocol and sequence parameters are summarized in Table 1.

### 2.3. Image Analysis

DKI data were analyzed using the prototype software developed in-house based on MATLAB 2013 (MathWorks, MA, USA). The pixel-wise ADC value, *D* value, and *K* value were fitted from multiple *b* values DKI datasets using a two-variable linear least-square method. The ADC value was fitted based on a monoexponential model by the following equation [10, 11]:

(1) $$\begin{array}{c} \ln\left(S\right)=\ln\left(S_{0}\right)-b\cdot ADC, \end{array}$$

where *S*_0_ is signal intensity for *b* = 0 and *S* is the measured signal intensity depending on the diffusion-weighting value *b*. The *D* and *K* values were fitted based on the non-Gaussian DKI model according to the following equation [10, 12]:

(2) $$\begin{array}{c} \ln\left(S\right)=\frac{\ln\left(S_{0}\right)-b\cdot D+1}{6b^{2}D^{2}K}, \end{array}$$

where *S* is the signal intensity depending on different *b* values, *S*_0_ is the signal intensity for *b* = 0, *K* is kurtosis, and *D* is true diffusivity. The kurtosis parameter quantified the deviation of water motion from Gaussian diffusion. *K* = 0 for perfect Gaussian diffusion, and a large kurtosis value indicates a marked deviation from the Gaussian distribution. The diffusivity is the diffusion coefficient corrected for non-Gaussian bias [3, 13]. The imaging datasets were analyzed by two experienced gastrointestinal radiologists (W and X with more than 10 years of experience in interpreting rectal MR images), who were blinded to the patients' clinical and pathological information independently. ROI was manually drawn along the border of the tumor on the parameter maps by the two observers. The T2-weighted images were used as a reference to maximally encompass the solid tumor and avoid the peripheral fat, visible necrotic or cystic areas, and distortion artifacts. In the current study, the ROI was drawn on each consecutive tumor-containing section, and all the parameters were measured by voxel using the whole-volume method. The lymph nodes were also assessed on the basis of conventional MRI images and DKI sequences by the two observers independently, based on the previously published criteria [14, 15], including size, border, and signal of the lymph node.

### 2.4. Statistical Analysis

The mean values of all the parameters measured by the two radiologists were used in the statistical analysis conducted using the statistical software SPSS 23.0 (IBM SPSS Statistics version 23.0, Armonk, NY, USA). *p* values < 0.05 were considered as statistically significant. All parameters were first tested by the Kolmogorov-Smirnov test for normality analysis and the Levene's test for variance homogeneity. Independent sample *t*-test was used to analyze and compare the parameters between pT1-2 and pT3-4. The receiver operating curve (ROC) was used to assess the diagnostic performance of the parameters, and the area under the receiver operating characteristic curve (AUC), sensitivity, and specificity was calculated. The cut-off value was calculated by maximizing the Youden's index. The intraclass correlation coefficient (ICC) was used to evaluate the stability of the parameters between the two observers.

## 3. Results

### 3.1. Patients and Histopathological Findings

Thirty-five patients underwent a low anterior resection, twelve patients underwent an abdominoperineal resection, and three patients underwent an extended resection. Among the 50 patients with tumors, 19 tumors were T1-2 (38%) and 31 were T3-4 (62%), according to the results of the surgical specimen analysis. Moreover, 53 lymph nodes were positive, according to the pathological results. MRI (DKI sequence with conventional sequences) showed 36 positive lymph nodes. The accuracy of DKI coupled with conventional sequences in identifying positive lymph nodes was 68%. The results are summarized in Table 2.

### 3.2. DKI Indices in Differentiating Different T Stages of Rectal Cancer

**Figure 1:**
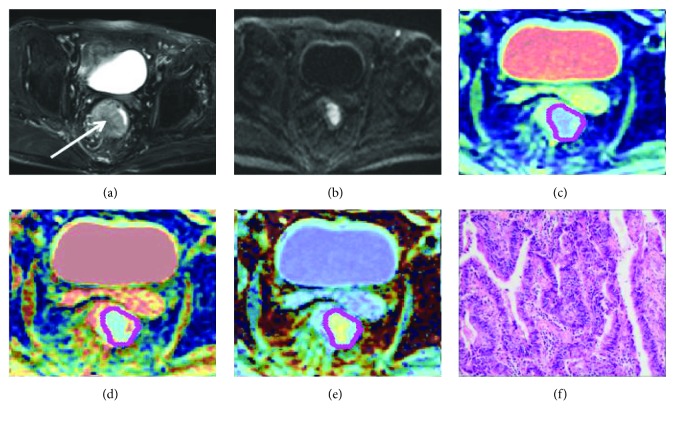
Images in a 65-year-old male show T2 stage in pathology. (a) Oblique axis T2WI, the white arrow shows the thickened rectal wall. (b) DKI shows a high signal of the thickened rectal wall. (c) ADC map, ADC-10th percentile value is 0.52 × 10^−3^ mm^2^/s. (d) Diffusivity map, *D*-10th percentile value is 0.72 × 10^−3^ mm^2^/s. (e) Kurtosis map, *K*-10th percentile value is 0.54. (f) The histological specimen shows moderately differentiated rectal adenocarcinoma (HE staining, ×40).

**Figure 2:**
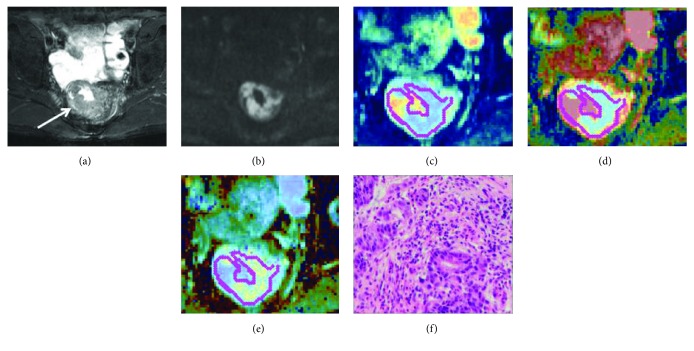
Images in a 61-year-old male show T2 stage in pathology. (a) Oblique axis T2WI, the white arrow shows the annular thickening mass of rectum. (b) DKI (*b* = 2000 s/mm^2^) shows a high signal of the thickened mass of the rectal wall. (c) ADC map, ADC-10th percentile value is 0.48 × 10^−3^ mm^2^/s. (d) Diffusivity map, *D*-10th percentile value is 0.67 × 10^−3^ mm^2^/s. (e) Kurtosis map, *K*-10th percentile value is 0.64. (f) The histological specimen shows moderately differentiated rectal adenocarcinoma (HE staining, ×100).

The histogram analysis of DKI parameters is shown in Table 3. Among the indices of ADC percentile and mean value, ADC-10th percentile and ADC-25th percentile were significantly higher in T1-2 than in T3-4 rectal cancer (the ADC values were 0.65 ± 0.08 × 10^−3^ mm^2^/s versus 0.58 ± 0.11 × 10^−3^ mm^2^/s and 0.73 ± 0.11 × 10^−3^ mm^2^/s versus 0.65 ± 0.11 × 10^−3^ mm^2^/s; *p* values were 0.035 and 0.024, resp.). In addition, among all the indices of *D* value, *D*-10th percentile and *D*-25th percentile were significantly higher in T1-2 than in T3-4 rectal cancer (the *D* values were 0.96 ± 0.19 × 10^−3^ mm^2^/s versus 0.84 ± 0.16 × 10^−3^ mm^2^/s and 1.15 ± 0.27 × 10^−3^ mm^2^/s versus 0.99 ± 0.18 × 10^−3^ mm^2^/s; *p* values were 0.017 and 0.044, resp.). No statistical difference was observed in all the kurtosis (*K* value) indices between early and late rectal cancer (Figures 1 and 2).

### 3.3. Performance of DKI Histogram Indices to Distinguish the Different T Stages of Rectal Cancer

**Figure 3:**
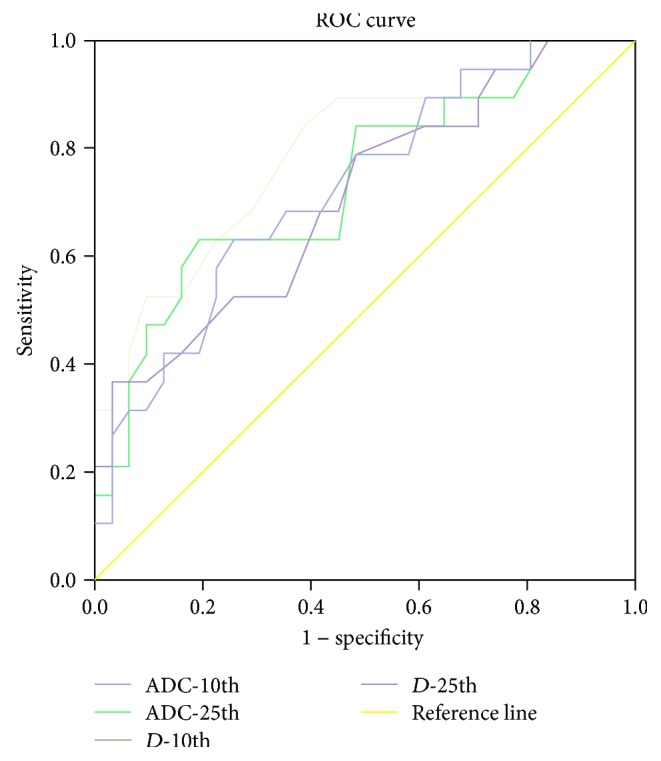
The ROC map of DKI parameters. *D*-10th percentile value had the largest area under the ROC curve (AUC 0.799), and the cut-off value, sensitivity, and specificity were 0.875, 84.2%, and 61.3%, respectively.

Among the DKI histogram indices, ADC-10th, ADC-25th, *D*-10th, and *D*-25th percentile values were significantly different between early and advanced rectal carcinoma. The *D*-10th percentile value had the largest area under the ROC curve (AUC 0.799), whose cut-off value, sensitivity, and specificity were 0.875, 84.2%, and 61.3%, respectively. The *D*-10th percentile value showed an 84.2% sensitivity. According to the results, the 25th percentile ADC value showed the highest specificity of 80.6%. The results were summarized in Table 4 and Figure 3.

### 3.4. Interobserver Agreement

The ADC value, *D* value, *K* value, and its histogram indices were consistent among the two interpreters. The interobserver agreement (ICC) of some histogram indices was shown in Table 5.

## 4. Discussion

DKI was first proposed by Jensen et al. [3] to account for the non-Gaussian diffusion property resulting from the microstructure complexity of tissues. DWI is based on the assumption that water molecule diffusion occurs in the biological tissues, which is Gaussian distribution. However, the water molecule diffusion is restricted in biological tissues due to the non-Gaussian distribution based on the microstructure complexity of tissues. The microstructure of the tumor tissue is complex and heterogeneous. The parameter diffusivity from non-Gaussian diffusion distribution theory could reflect the diffusion of water molecules in the tumor tissue. The advanced rectal cancer has a large volume and deep infiltration, and the solid ingredients and microvascular may enlarge the tumor cell density. As a result, the tissue of advanced rectal carcinoma is more complex and heterogeneous in microstructure than those in the earlier stages. The more complex the tissue microstructure, the more restricted diffusion of water molecules, thereby indicating a lower *D* and ADC values and higher *K* value. In our results, the *D* and ADC values were statistically higher in early rectal cancer than in the advanced group, and the *K* value was lower in both groups, corresponding to the theory of water molecule diffusion in the tissue as described above. Some previous studies reported that the ADC value was much higher in advanced rectal cancer than in the early stage [16, 17]. However, there also existed contradictory results in some studies [18, 10, 19] that the ADC value could not distinguish the advanced rectal cancer from an early stage. And in our article, the AUC, sensitivity, and specificity of *D*-10th were 0.799, 84.2%, and 61.3%, respectively, in differentiating early and advanced rectal cancer. As to ADC-10th, the AUC, sensitivity, and specificity were 0.699, 78.9%, and 51.6%, respectively. Thus, the diagnostic performance of *D*-10th is superior to the ADC-10th in assessing T stages of rectal cancer based on the theoretical characteristics of DKI. So, DKI can more accurately reflect the diffusion of water molecules of biological tissues. Although no statistical differences in *K* value were observed between T1-2 and T3-4, it showed the trend to the higher *K* value with the higher T stage of rectal cancer. And similar result had been reported [10]. DKI quantitative indices were more objective and stable than traditional MRI in rectal cancer. DKI is a valuable supplement method to traditional MRI in the diagnosis of rectal cancer.

In this study, we used whole-tumor volume histogram analysis, which analyzed the smallest unit of a voxel in every section that contained lesions. The current results demonstrated that both the 10th and 25th percentile values of ADC and diffusivity were lower in T3-4 than in T1-2 rectal cancer, albeit the differences were not statistically significant. In advanced rectal cancer, the necrotic cystic area was primarily in the center of the tumor. In the necrotic cystic area, the membrane tumor cell may lose its integrity. Consequently, the diffusion of water molecules was not restricted, indicating the high value of ADC and diffusivity. Furthermore, on the edge of the tumor, the tumor cell hyperplasia is rather active, and the tumor cell atypia is distinct; the tumor tissue microstructure is complex, and the water molecule diffusion is complex and restricted. Therefore, the values of ADC and diffusivity are lower on the edge of tumor mass that might represent the 10th and 25th percentile values according to the histogram analysis. A similar study also demonstrated that the *D*-25th percentile might represent the area of lower *D* value in a part of the heterogeneous tumor mass [20]. In the rectal cancer TNM staging system, the depth of tumor invasion of the intestinal wall is an evaluation criterion for the T stage. Thus, the tumor cell hyperplasia is active on the edge of the mass, and this explained the difference in the 10th and 25th percentile values of ADC and diffusivity in T1-2 and T3-4 in rectal cancer. Thus, the histogram analyses could be deemed valuable in assessing the preoperative T stage of rectal carcinoma.

Inside the involved lymph node of rectal cancer patients, the normal tissue is replaced by tumor cells, followed by an increase in the cell density. Therefore, the microstructure is complex, and the diffusion of water molecule is restricted, thereby indicating that the lymph node is distinctly shown as a high signal in the DKI sequence. The precise staging of lymph nodes is critical to the choice of clinical treatment, thereby necessitating the improvement of the accuracy of lymph node staging. Among the current methods of examination, MRI has a greater advantage in lymph node staging. MRI has the advantage of multiple sequences and parameters, including conventional sequences, diffusion-weighted imaging (DWI), and the dynamic contrast-enhanced sequence. However, the accuracy of the method failed to satisfy the clinical need. The study by Doyon et al. [21] showed that the sensitivity, specificity, and accuracy of conventional MRI in evaluating the lymph node staging were 94%, 13%, and 34%, respectively. Furthermore, the studies by Maier et al., Gagliardi et al., and Kim et al. [22–24] demonstrated that the accuracy of traditional MRI in assessing lymph node staging was 43%, 69%, and 72%, respectively. Therefore, the traditional MRI in assessing lymph node staging was not adequate, and the results of different studies varied widely. Recently, DWI has frequently been used in assessing the stage of lymph nodes; however, the traditional method is yet controversial. Curvo-Semedo et al. [18] and Cho et al. [25] showed that the ADC value was advantageous in differentiating the involved lymph node, and the accuracy of the latter was 70%. Nevertheless, other studies yielded opposite, contradictory result, showing that the ADC value was pointless [14, 21, 26]. In the current study, DKI coupled with the traditional sequences had an accuracy of 68% in confirming the positive lymph nodes in rectal cancer, which was similar to the other findings. The present study did not measure the values of quantitative DKI parameters while assessing the lymph nodes. Taken together, the current tools of evaluating the rumor are not adequate in distinguishing the involved lymph nodes; however, the multiple *b* value DKI sequences exhibited a superior potential, although more large sample studies are imperative to substantiate the value of DKI.

In worldwide, DKI is an interesting technique especially in breast cancer, prostate cancer, and cervical cancer. As in rectal cancer, DKI focused on histological grade and the evaluation of neoadjuvant chemoradiation in which DKI shows good performance and the potential application value in rectal cancers. In our study, the major finding of this study is that we used whole-volume histogram analysis to evaluate the value of DKI parameters in preoperative staging and heterogeneity of rectal cancer. The results showed that *D*-10th percentile was mostly accurate in distinguishing T1-2 from T3-4, which indicates that *D* value may serve as a potential biological indicator to evaluate the preoperative staging of rectal cancer noninvasively. Also, the study has some limitations. Firstly, this was a retrospective study, and hence, a selection bias would be inevitable. Secondly, the sample size was relatively small. Therefore, a large sample and multicenter study are essential to further verify the value of DKI.

## Figures and Tables

**Table 1:** Imaging protocol parameters and sequences.

Parameters	T1WI	T2WI	DKI
Imaging direction	Oblique axis	Oblique axis	Oblique axis
TR (ms)	700	3770	4900
TE (ms)	12	101	87
PAT	2	2	4
FOV (mm^2^)	280 × 280	200 × 200	280 × 280
Matrix	128 × 128	128 × 128	128 × 128
Slice thickness (mm)	4	4	4
Number of sections	20	20	20
*b* value (s/mm^2^)			200, 500, 1000, 1500, and 2000

**Table 2:** Demographic and clinicopathological characteristics.

Characteristic	Result
Gender	
Male	27 (54%)
Female	23 (46%)
Mean age (y)	57.7
Tumor location	
Low rectum	28 (56%)
Mid rectum	19 (38%)
High rectum	3 (6%)
T stage	
T1-2	19 (38%)
T3-4	31 (62%)
Number of p-LN+^∗^	53
Number of MRI-LN+	36 (68%)

Note: p-LN+^∗^ indicates pathological positive lymph node.

**Table 3:** Comparison of histogram imaging indices between T1-2 and T3-4.

Histogram variable	T1-2	T3-4	*p* value
ADC-mean	0.88 ± 0.17	0.82 ± 0.13	0.153
ADC-10th	0.65 ± 0.08	0.58 ± 0.11	**0.035**
ADC-25th	0.73 ± 0.11	0.65 ± 0.11	**0.024**
ADC-50th	0.84 ± 0.15	0.77 ± 0.13	0.066
ADC-75th	1.02 ± 0.26	0.93 ± 0.190.9	0.213
ADC-90	1.18 ± 0.34	1.14 ± 0.28	0.674
*D*-mean	1.50 ± 0.38	1.35 ± 0.27	0.153
*D*-10th	0.96 ± 0.19	0.84 ± 0.16	**0.017**
*D*-25th	1.15 ± 0.27	1.00 ± 0.8	**0.044**
*D*-50th	1.42 ± 0.38	1.25 ± 0.26	0.088
*D*-75th	1.84 ± 0.63	1.63 ± 0.43	0.21
*D*-90th	2.14 ± 0.63	2.05 ± 0.57	0.622
*K*-mean	0.74 ± 0.10	0.82 ± 0.26	0.207
*K*-10th	0.48 ± 0.11	0.47 ± 0.09	0.924
*K*-25th	0.61 ± 0.12	0.62 ± 0.17	0.899
*K*-50th	0.75 ± 0.12	0.81 ± 0.16	0.167
*K*-75th	0.88 ± 0.11	0.99 ± 0.29	0.123
*K*-90th	0.99 ± 0.10	1.18 ± 0.66	0.223

Data are expressed as means ± standard deviations. Diffusivity and ADC values are expressed as mm^2^/s. *p* < 0.05 is statistically significant.

**Table 4:** ROC analysis results of parameters.

Histogram variable	AUC	Cut-off value	Sensitivity (%)	Specificity (%)
ADC-10th	0.699	0.6	78.9	51.6
ADC-25th	0.737	0.71	63.2	80.6
*D*-10th	0.799	0.875	84.2	61.3
*D*-25th	0.735	1.06	63.2	74.2

**Table 5:** Interobserver agreement (ICC) for variable measurement.

Histogram variable	ICC (95% CI)
ADC-mean	0.953 (0.918–0.973)
ADC-10th	0.849 (0.734–0.914)
ADC-25th	0.893 (0.811–0.939)
*D*-mean	0.951 (0.913–0.972)
*D*-10th	0.944 (0.901–0.968)
*D*-25th	0.943 (0.900–0.968)
*K*-mean	0.940 (0.895–0.966)
*K*-10th	0.923 (0.864–0.956)
*K*-25th	0.880 (0.788–0.932)

## Data Availability

The data used to support the findings of this study are available from the corresponding author upon request.
